# Trauma-Focused Group-Based Psychological Program for Children and Adolescents Following a Terrorist Attack on a School in Afghanistan: An Uncontrolled Pre-Post Follow-Up Study

**DOI:** 10.1007/s40653-026-00911-5

**Published:** 2026-05-22

**Authors:** M. Ali Amiri, Ian George Barron, Gerhard Andersson

**Affiliations:** 1 Department of Behavioral Sciences and Learning, Linköping University, Linköping, SE-58183 Sweden; 2 Faculty of Psychology and Educational Sciences, Kabul University, Kabul, Afghanistan; 3 Center for Youth Engagement, University of Massachusetts, Massachusetts 01003 Amherst, USA; 4 Department of Biomedical and Clinical Sciences, Linköping University, Linköping, Sweden; 5 Department of Clinical Neuroscience, Psychiatry Section, Karolinska Institutet, Stockholm, Sweden

**Keywords:** Terrorist attacks, Trauma-focused programs, PTSD, Afghan children, Hazara people, School bombing

## Abstract

The current study assessed the effectiveness and acceptability of the Children and Grief (C&G) program in mitigating symptoms of post-traumatic stress disorder (PTSD) among children and adolescents aged 10–17 years, following a terrorist attack on a school in Afghanistan. A total of 204 Afghan (Hazara) children and adolescents exposed to the Sayed al-Shuhada school terrorist attack participated in the C&G group-based intervention. Seven weekly sessions of 90 min were delivered at an established psychosocial center within the school building in Kabul. The sessions focused on teaching coping skills for PTSD. Outcomes were measured using the Children’s Revised Impact of Events Scale (CRIES-8) and the General Health Questionnaire (GHQ-28) at post-intervention and one-month follow-up. A repeated-measures analysis of variance (ANOVA) was used on quantitative data. Both written and verbal feedback were collected from participants, and the subjective experience of program delivery was explored with 10 group facilitators. The results indicated a large effect size, suggesting a significant reduction in PTSD symptoms and an increase in psychological wellbeing. These findings suggest that the C&G program shows promise for treating PTSD in children and adolescents within emergency settings. Randomized controlled trials, exploration of participants’ response style, and longitudinal studies are warranted.

## Introduction

Afghanistan has gone through a turbulent socio-political change in its history. As a result of over four decades of war and political upheaval, the people have suffered from a combination of armed conflict, protracted poverty, and social injustice (Panter-Brick et al., 2009). A series of violent conflicts since 1979, starting with the Soviet Union’s invasion, have made millions of Afghans suffer psychologically (Alemi et al., 2023; Mohd Saleem et al., 2021). The country’s inhabitants across generations have been exposed to systematic violence resulting in grave human rights violations and mass displacement (Silove & Ventevogel, 2022). The deteriorating effects of unrelenting conflict not only affected the population in the country but also Afghan refugees (Alemi et al., 2023).

Throughout the history of the conflict, schools have been one of the primary targets of contention between the armed groups (World Bank, 2009). Afghanistan’s education system has witnessed various types of attacks, including arson, bomb explosions in or near schools buildings, the murder of teachers and students, and warnings for students not to attend school (World Bank, 2009; Human Rights Watch [HRW], 2006). Evidence indicate that girls’ education has been particularly targeted. Between 2006 and 2008, attacks on girls’ schools accounted for 40% of all attacks on schools in Afghanistan including boys’ schools, mixed schools, and girls’ schools (World Bank, 2009). Various reasons have been cited as contributing to these attacks. The World Bank’s (2009) report highlights that opposition groups perceive schools as government entities and target them to disrupt the relationships between the government and people. Furthermore, rigid cultural and religious norms surrounding women’s education in Afghanistan, negative gender stereotypes, and general security issues (Shir Mohammad, 2018), have likely created conditions that make girls’ education particularly vulnerable to violence.

On 8 May 2021, a terrorist attack occurred at Sayed al-Shuhada high school in western Kabul, Afghanistan. A car bomb exploded between two main gates followed by another blast only five minutes later, and yet another one about three minutes after that. The explosions were incredibly powerful, causing extensive damage to the school building and nearby residential and public properties. Initially, reports indicated that 55 people were killed and over 150 injured (Neely, 2021). However, the final total reached 90 killed and 240 injured (Gibbons-Neff, 2021). Many children and adolescents suffered severe injuries including the loss of limbs or soft tissues damage, and some were scorched and disfigured. Although the school provides education for boys and girls in three different shifts, the terrorist attack occurred just as the girls left the school compound, indicating that girls and their teachers were the main targets. Many survivors witnessed the horror and suffering of the attack.

The bombing was recognized as a targeted terrorist attack on a specific ethnic group as all the victims belonged to the Hazara ethnic group (Gibbons-Neff, 2021; Neely, 2021). Throughout the history of Afghanistan, minority religious and ethnic groups have faced discrimination and victimization (Silove & Ventevogel, 2022), leading to a higher risk of mental health problems within these groups (Alemi et al., 2023). However, the Hazaras, an ethnic group that is predominantly Shia Muslim, have endured significant discrimination, mass killings, and other serious abuses by successive Afghan rulers (Karimi & Nazari, 2025; HRW, 2022), and non-state actors (Ashrafian, 2023) throughout the history of the country. Research on the Hazara community’s mental health indicates that successive experiences of severe violence, brutal killings, and loss of family members is a leading cause of high levels of Post-Traumatic Stress Disorder (PTSD; 68.2%) and depression (51.7%) within this community (Zarak et al., 2020).

Direct exposure to a bomb blast is associated with a higher level of probable PTSD and psychological distress in children affected by a bombing incident (McDermott et al., 2013). Studies show that at least 28–35% of people exposed to a terrorist attack may develop PTSD, with a higher risk among women and young individuals (Lee et al., 2002). In war contexts where exposure to various traumas is constant, physical and psychological symptoms are more pronounced (Murthy & Lakshminarayana, 2006). In Afghanistan, Ahmadi et al. (2022a) studied a sample of 376 adolescents and found that 106 individuals (28.2%) were at significant risk of psychiatric problems, 194 (51.6%) met criteria for probable PTSD, 184 (48.9%) for depression, and 170 (45.2%) for anxiety. Younger age was associated with poorer mental health, and female participants had a higher probability of having psychiatric disorders. Individuals who are exposed to prolonged traumatization due to ongoing combat, genocide campaigns, and violence may also develop a complex form of traumatic reactions known as Complex PTSD (C-PTSD) characterized by disturbances in self-organization symptoms in addition to PTSD symptoms (Maercker et al., 2022). C-PTSD is associated with both childhood and adulthood prolonged trauma and the severe interpersonal nature of trauma (Palic et al., 2016).

Research indicates that in conflict settings and Low- and Middle-Income Countries (LMICs), various forms of violence as significant risk factors for mental health predominately affect children and adolescents (Abrahams & Nöthling, 2024; Kieling et al., 2011). In these contexts, despite a need for mental health services, resources are scarce to meet those needs (Giebel et al., 2024). Resources are especially limited for children and adolescents despite being a large portion of the population in LMICs and the importance of mental health in this period of life (Bolton et al., 2023). Inadequate mental health services in most of these countries are due to limited public funding, insufficient mental health workers (World Health Organization, 2025), as well as issues beyond funding such as abuse, discrimination, and stigma against mental health, and cultural stereotypes that contribute to poor mental health (Alloh et al., 2018). Besides socioeconomic conditions, terrorism influences healthcare services in LMICs by exacerbating morbidity, mortality, and treatment delays (Adebusoye et al., 2024). Active conflict and political instability can also be considered major obstacles to establishing a sustainable system of mental health support (Mohamed et al., 2021) and continued research. The current study was also disrupted by the collapse of the former Islamic Republic of Afghanistan government on August 15, 2021, which led to a pause in the study and potentially increased distress and uncertainty among participants. As a result, the team had to repeat assessments, form new groups, and cope with the loss of some initial participants.

Multiple therapeutic approaches have been developed to address trauma-related psychopathology in children and adolescents. Some evidence-based approaches include Trauma-Focused Cognitive Behavioral Therapy (TF-CBT; Cohen et al., 2012), Narrative Exposure Therapy (KIDNET; Schauer et al., 2017), Cognitive Behavioral Intervention for Trauma in Schools (CBITS; Jaycox et al., 2012), and Prolonged Exposure Therapy (PET; Foa et al., 2009). These approaches have also been used in disaster contexts. For example, Jaycox and colleagues (2010) implemented CBITS following Hurricane Katrina, TF-CBT was implemented to youth affected by the World Trade Center disaster (CATS Consortium, 2010), and KIDNET was used with refugee children in exile who had been traumatized by organized violence (Ruf et al., 2010). However, these treatments were delivered by trained clinicians in post-disaster and non-active conflict contexts.

Since war and active conflicts can affect large numbers of children and adults (Alemi et al., 2023), children may not only experience a single direct traumatic incident but also suffer from other war-related events such as displacement, school disruption, and loss of friends and relatives (Yule et al., 2013). Therefore, some programs based on these evidence-based approaches have been developed and adapted to the context of war and large-scale emergencies, where needs are high and resources are scarce. These programs have the potential to be delivered in groups and by non-mental health professionals or paraprofessionals as “low-intensity interventions” (Sijbrandij et al., 2020). For example, the Teaching Recovery Techniques (TRT) program, derived from TF-CBT, was delivered by school counselors after training to 133 children in Nablus, Palestine (Barron et al., 2013). Similarly the Writing for Recovery program, based on narrative therapy techniques, was delivered by teachers to bereaved Afghan adolescent refugees in Iran (Kalantari et al., 2012). In addition, a school-based group psychotherapy program focused on trauma and grief was delivered by school counselors to war-exposed adolescents in Bosnia (Layne et al., 2008).

Given that the students potentially suffered from both the loss of their classmates and post traumatic symptoms following the terrorist attack on their school in a protracted conflict context, the Children and Grief (C&G) program (Dyregrov et al., 2011) was deemed suitable to their needs and the context. The C&G program is based on cognitive behavioral theory and was specifically developed for war settings by the Children and War Foundation (Yule et al., 2013). The program aims to reduce both complicated grief and PTSD symptoms in children and adolescents through psychoeducation and the teaching of coping skills (Dyregrov et al., 2011). The PTSD component of the C&G program focuses on normalizing trauma responses and addressing trauma-related intrusions, hyperarousal, and avoidance symptoms through understanding of emotions, relaxation skills, and systematic desensitization, and the grief component teaches children and adolescents how to regulate grief reminders and adapt to living with loss over time (Barron & Abdallah, 2017; Dyregrov et al., 2011).

Following the Inter-Agency Standing Committee (IASC) guidelines on mental health and psychosocial support (MHPSS) in emergencies (IASC, 2007), and considering the large number of affected students, the C&G program was selected as a structured, scalable, group-based intervention that can be implemented by trained non-mental health professionals. The C&G manual had previously been culturally adapted for adolescents who experienced traumatic grief in Afghanistan as part of a master’s thesis by the first author (unpublished).

The C&G program has been implemented and evaluated in similar settings, for example in occupied Palestine, where there has been enduring war, violent military occupation, and terrorist attacks. The manual has been found effective in reducing PTSD, complicated grief, and depression among children and adolescents (Barron & Abdallah, 2017).

Due to the pressing needs of traumatized children following the terrorist attack on Sayed al-Shuhada school and the necessity for scalable, evidence-based, and culturally adapted psychological interventions for children and adolescents in Afghanistan that address both PTSD and psychological distress related to loss, the current pilot sought to implement the C&G program. In line with the IASC guidelines, which emphasize that emergencies require multi-layered and multisectoral MHPSS responses (IASC, 2007), the C&G program was implemented as part of a multi-layered emergency response to the terrorist attack on Sayed al-Shuhada school in western Kabul. The emergency response included specialized psychological assessment, treatment and referral, child protection case management, life-saving medical assistance, back to school campaigns and capacity building training for local residence on psychological first aid and emergency response.

The aim of this pilot study was to implement a trauma and loss-specific program designed to teach children coping skills to overcome psychological distress resulting from the terrorist attack. We aimed to investigate the potential of the C&G program in reducing the symptoms of PTSD and enhancing psychological wellbeing at post intervention and one month follow-up.

## Method

### Research Design

The uncontrolled study employed a pre-post and follow-up at one month design. Assessments were conducted by trained psychology graduates and students in the final year of their psychology training, who were blinded to the aims and hypotheses of the study. Considering the nature of the terrorist attack and the highly sensitive emergency setting, special measures were considered to prepare the groundwork for research and intervention. Initially, two psychological service centers in Kabul, located at significant distances from the targeted school, were contracted to provide a safe and friendly environment for the students while receiving psychological treatment. Participants were transported to the service centers and back home. However, following the collapse of the former Islamic Republic of Afghanistan government on August 15, 2021, when the intervention had not yet started but the assessment had partly been conducted, the contracted centers were closed. Therefore, the operation continued with the establishment of a psychosocial center and a medical emergency room equipped with primary health facilities inside the Sayed al-Shahada school premises. Students participated in the intervention on a weekly basis schedule at the center.

### Ethical Considerations

This study was conducted in accordance with the ethical standards outlined in the Declaration of Helsinki. The full emergency response was approved through a joint statement signed and issued by an action committee consisting of representatives from the Islamic Republic of Afghanistan government, international child protection organizations, academics, the school’s management committee, and the community’s representatives (*Shura*- a group of local people elected by the residents who are responsible for handling their communities’ issues). The study was approved for data analysis by the Swedish ethics review authority (Etikprövningsmyndigheten; 2024-04763-01).

Since the attack was considered a targeted attack on a specific ethno-religious group (the Hazaras), the entire intervention had to be conducted in a sensitive manner. Community engagement was ensured by involving community figures and representatives in coordination and access to participants. Written informed consent was obtained from participants and their legal guardians, ensuring their understanding of the intervention’s purpose and the voluntary nature of participation. Special care was taken to maintain the confidentiality and anonymity of all participants throughout the study, and the protocol was approved by the Faculty of Psychology and Educational Sciences, Kabul University, Kabul, Afghanistan.

### Participants and Procedure

Participants were required to have been exposed to the terrorist attack, meet the DSM-5 Criterion A for PTSD, and score at or above the cutoff on the Children’s Revised Impact of Event Scale (CRIES-8; score ≥ 17). Eligible participants were children and adolescents aged 10–17 years, from the targeted school in western Kabul, Afghanistan, who met the inclusion criteria for the group intervention. In total, 288 students (235 girls, 53 boys) were assessed for eligibility. Seventy-eight students (74 girls, 4 boys) were excluded due to severe or complex physical or mental health conditions, including life-threatening injuries or suicidality. One participant (girl) was excluded due to scoring below the CRIES-8 cutoff, and five participants (4 girls, 1 boy) were excluded because they could not be reached following the collapse of the government.

**Fig. 1:**
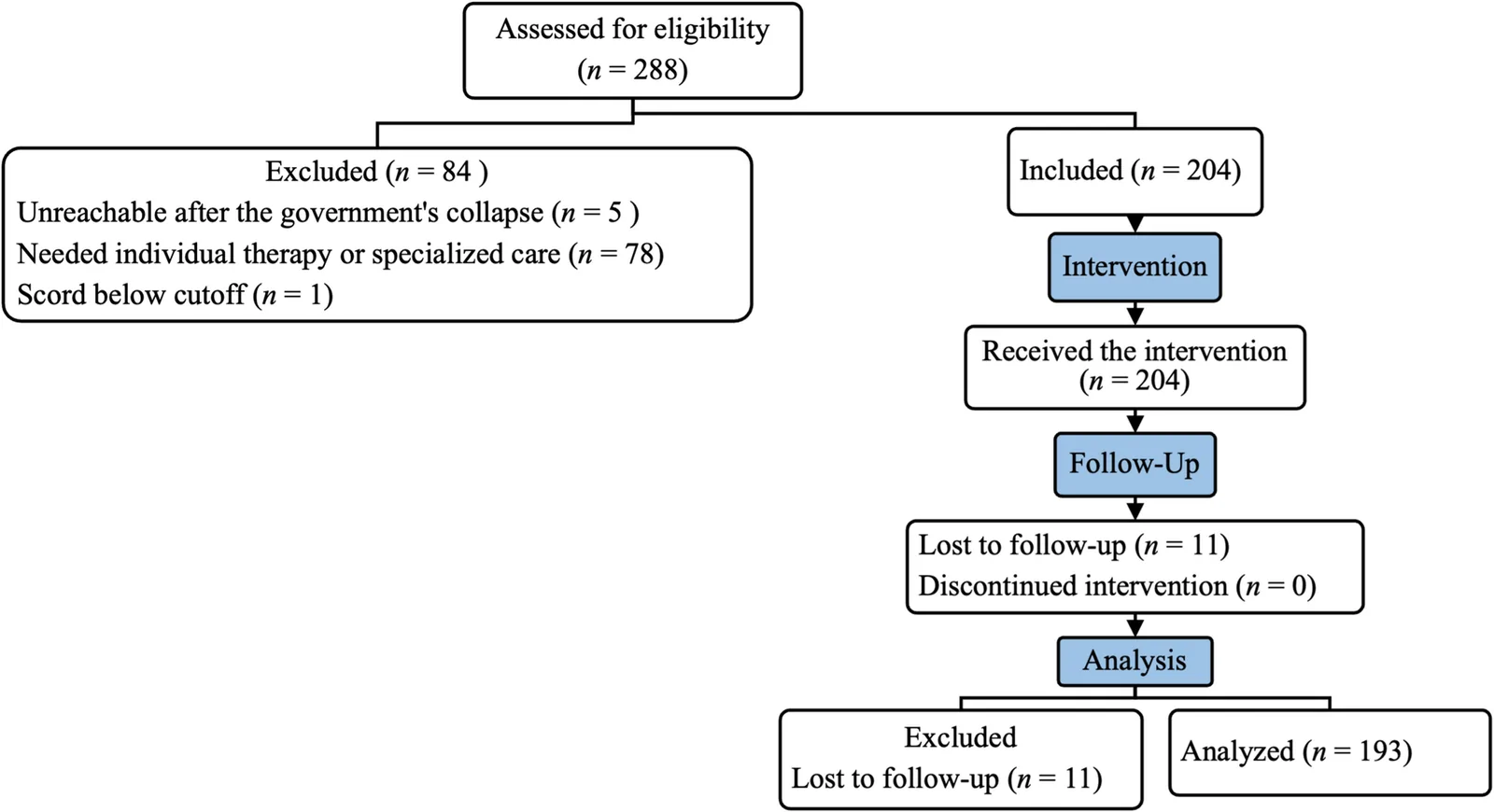
Participant flow chart

Ineligible participants were offered one-to-one psychotherapy (56 girls, 4 boys) or a combination of individual therapy and referral to psychiatric care (18 girls). A total of 204 participants were included in the group intervention. Eleven participants (7 girls, 4 boys) were lost to follow-up due to security situation and relocation. The participant flow chart is presented in Fig. 1.

Participants were allocated to groups, each consisting of 12 individuals. They were classified into three priorities based on the severity of their problems. Participants with high scores in pre-intervention screening and reporting severe intrusion and avoidance symptoms were given priority and received the program first. Single gender groups were formed homogeneously based on participant’s availability, gender, age, and priority level.

Questionnaires were administrated in a paper-based format, and data were manually retrieved from the papers.

The final data set included 193 participants. The mean age was 12.44 years (range 10–17 years, *SD* = 1.23) and there were 149 girls and 44 boys. The participants were pupils in either primary (86.5%) or secondary (13.5%) levels at Sayed-al Shuhada school.

### Program Presenters

The group intervention was presented by ten facilitators: seven were final-year undergraduate psychology students, and the remaining three held bachelor’s degrees in psychology. Among the facilitators, three were female and seven were male. All had previously completed a professional course in Cognitive Behavioral Therapy (CBT) and underwent a specific training for this study. The training covered the implementation of the C&G program, psychological and child protection needs assessment in emergency settings, ethical principles in counseling and psychotherapy as well as child rights and child safeguarding. The training was conducted by a clinical psychologist and university lecturer (the first author), and a child protection specialist (both Afghan professionals). Following the training and a simulated implementation of the intervention, the trained facilitators were deployed to conduct the intervention.

### Program

The program “Children and Grief: Teaching Life Skills” was developed by the Children and War Foundation (Dyregrov et al., 2011). The program is based on cognitive behavioral theory and aims to reduce post-traumatic stress symptoms and symptoms of complicated grief. The core program for C&G, *TRT* was designed to reduce posttraumatic stress symptoms through psychoeducation and the teaching of coping skills (Barron & Abdallah, 2017).

The C&G program is implemented in seven sessions. The first session is focused on the normalization of the trauma response. This session introduces the idea of creating a toolbox of strategies, an imagery safe place, a feeling thermometer for measuring the intensity of the emotions and a memory folder. Sessions two to four focus on post-traumatic stress symptoms and address intrusions, hyper-arousal, and avoidance. In working with intrusions, the program introduces visual imagery and dual attention techniques. Teaching relaxation techniques, breathing, mindfulness and coping statements techniques are used to address arousal symptom. For avoidance symptoms, the program introduces procedures and techniques such as regulating grief and traumatic event reminders, distraction, and sharing experiences. Sessions five and six focus on understanding and coping with feelings. The techniques in these sessions include postponing worry and sadness, positive self-statements, and strong thoughts, working on sleep problems (e.g., working on intrusive dreams and nightmares) and participation in the funeral. The final session teaches children and adolescents how to live with loss over time through meaning making, continuing relationships, and expecting the future.

Some minor modifications were made to the protocol to adapt the content and techniques to the context, considering cultural practices related to death and mourning. Although, the original program addresses post-traumatic stress symptoms and complicated grief simultaneously, this intervention maintained a primary focus on post-traumatic stress symptoms and coping strategies rather than grief-related problems. This decision was based on the absence of reported losses of siblings or close relatives among participants. While some participants reported the loss of a classmate or someone they knew at the school, but the nature of their relationship to the deceased was unclear. In addition, the screening data indicated that intrusive and avoidance symptoms were the predominant difficulties reported by participants.

Related to the grief component, attending a funeral ceremony or performing a symbolic funeral as they are explained in the program was unfamiliar to the participants. Culturally, children and adolescents in Afghanistan are not typically involved in funeral ceremonies, and discussions about deceased individuals with children are often discouraged. Instead, a local and culturally familiar practice was adapted, in which group participants gathered and collectively prayed for all students who had died, wishing that they rest in peace. Participants found the practice positive.

The Persian/ Dari translation of the C&G program was used and the adaptation to the context was done by the first author. Group activities were carried out by two trained group facilitators as main and co-facilitators. The intervention spanned over seven weeks, with participants attending one session per week. Each session lasted 90 min and included a break after 45 min, during breaks participants were provided with nutritious snacks and free time to relax.

### Outcome Measures

The Persian versions of the Children’s Revised Impact of Event Scale (CRIES-8) and the General Health Questionnaire (GHQ-28) were implemented two weeks prior to the intervention, at the end of the intervention’s last session and at a one-month follow-up after the last delivery session of the C&G program. The CRIES-8 is a measure of post-traumatic stress for children aged 8 years and older, who are able to read independently (Perrin et al., 2005). The questionnaire consists of 4 items measuring intrusive thoughts, memories and images, and 4 items measuring avoidance of thoughts and reminders associated with the traumatic event. The questionnaire has 8 items on a 4-point scale (from “not at all” to “often”) and a total score of 17 or above indicates a likely PTSD diagnosis (Perrin et al., 2005). CRIES- 8 subscales show a good internal consistency (Cronbach’s alpha 0.82 for intrusion and 0.74 for avoidance; Dyregrov et al., 1996). The internal consistency for the Persian version of CRIES-8 in a sample of bereaved Afghan adolescents was α = 0.74 (Sajadian, 2012).

The GHQ-28 is a screening tool used to detect minor psychiatric disorders in the general population, within community and non-psychiatric clinical settings (Sterling, 2011). The questionnaire consists of four subscales measuring somatic symptoms, anxiety and insomnia, social dysfunction, and severe depression (Goldberg & Hillier, 1979). The questionnaire can be scored from 0 to 3 for each response with a total possible score ranging from 0 to 84. Numerous studies have investigated reliability and validity of the GHQ-28 in various clinical and non-clinical populations. In the Persian version, the clinical cutoff of this questionnaire was 24, and a Cronbach’s alpha of 0.97 was reported (Ebrahimi et al., 2007). In this study GHQ-28 was used as a measure of psychological wellbeing.

### Data Collection

Data collection consisted of three key phases. The data were collected and stored at Children in Crisis/Street Child (an international non-governmental organization in Kabul).

### Pre-Intervention Assessment

Prior to the start of the intervention, a pre-screening was employed. Participants completed two validated questionnaires, CRIES-8 and GHQ-28, to report trauma-related symptoms and psychological wellbeing. A short form of socio-demographic questionnaire was also administered. The questionnaires’ administration was complemented by a semi-structured interview. The interview was carried out by trained group facilitators to make sure that participants met the inclusion criteria.

### Post-Intervention Assessment

In the last session of the intervention, the CRIES-8 and GHQ-28 measures were completed again. This post-intervention assessment aimed to evaluate changes in psychological wellbeing and PTSD symptoms severity. Participants’ oral and written feedback about the program design and its effects were also collected at the last session of the intervention, wherein the group participants were asked to reflect on their experience of receiving the C&G intervention and its helpfulness and sensitivity to their language and culture.

### Follow-Up Assessment

One month after the intervention, participants were contacted to complete the CRIES-8 and the GHQ-28 questionnaires again. This follow-up assessment allowed for the examination of sustained changes in psychological wellbeing and PTSD symptoms over time.

### Data Analysis

Data were analyzed using IBM SPSS Statistics (Version 30.0). In the data cleaning process, no outliers were detected and the data were normally distributed for other variables except for the avoidance variable that did not meet the assumption of normality. The two-step data transformation for normality (Templeton, 2011) was used. Statistical analyses using repeated-measures analysis of variance (ANOVA) with Huynh-Feldt correction for degrees of freedom were performed. Pairwise comparisons were performed using t-tests with Bonferroni correction to assess the significance of changes in the measured variables. Effect sizes for the repeated-measures ANOVA were evaluated using partial eta squared for overall effects, and Cohen’s *d* for comparing specific paired groups. Analysis primarily focused on within-group changes across assessment phases (pre-intervention, post-intervention, and follow-up). Given that the PTSD subscales data (intrusion and avoidance) were missing for 30 participants, subscale analyses were conducted on 163 participants, and the results are reported accordingly.

## Results

### PTSD Symptoms

Following the delivery of the C&G program, participants showed a significant reduction in overall post-traumatic stress symptoms. Means and standard deviations for the outcome measures at pre-, post- intervention and one month follow-up are presented in Table 1. With PTSD, the analysis indicated a significant effect of the C&G program over time, *F*(1.87, 359.32) = 964.85, *p* < .001, η_p_^2^ = 0.83. Pairwise comparisons indicated a significant difference between pre-intervention and post-intervention, *t*(192) = 30.11, *p* < .001, *d* = 2.16. This significant difference persisted at one-month follow-up with t(192) = 9.16, *p* < .001, *d* = 0.66.

**Table 1:** PTSD symptoms (CRIES-8), intrusion, avoidance, and psychological wellbeing (GHQ-28) - means and standard deviations

Measures	Pre-intervention		Post-intervention		One-month follow-up	
	M	SD	M	SD	M	SD
CRIES-8	31.60	5.25	14.17	6.17	10.04	5.22
Intrusion	14.71	3.66	6.08	3.68	4.02	2.50
Avoidance	16.83	2.25	8.01	4.70	5.83	3.62
GHQ-28	44.66	11.80	28.31	8.94	22.65	6.94

Abbreviations: *CRIES-8* Children’s Revised Impact of Event Scale 8-items, *GHQ-28* General Health Questionnaire 28-items

A significant intervention effect was observed for intrusion symptoms, *F*(1.80, 293.12) = 508.69, *p* < .001, η_p_^2^ = 0.75. Pairwise comparisons indicated significant reductions in intrusions from pre-intervention to post-intervention, *t*(162) = 20.96, *p* <. 001, *d* = 1.64, and from post-intervention to one-month follow-up, *t*(162) = 6.49, *p* < .001, *d* = 0.50. Similarly, the intervention showed an overall significant effect on avoidance symptoms across assessment points, *F*(1.88, 305.70) = 481.11, *p* < .001, η_p_^2^ = 0.74, with significant decrease from pre-to post-intervention, *t*(162) = 22.33, *p* < .001, *d* = 1.74, and from post-intervention to one-month follow-up, *t*(162) = 5.38, *p* < .001, *d* = 0.42.

### Psychological Wellbeing

With psychological wellbeing, a significant difference was found across the three assessment points, *F*(1.77, 344.30) = 391.80, *p* < .001, η_p_^2^ = 0.67. The scores significantly differed between pre-intervention and post-intervention, *t*(192) = 20.75, *p* < .001, *d* = 1.49, and post-intervention and one-month follow-up, *t*(192) = 8.07, *p* < .001, *d* = 0.58. The statistically significant decreasing trend in GHQ-28 scores demonstrates a sustained improvement in participants’ psychological wellbeing over time. None of the interactions between treatment and age, school level, or gender were significant (all *p*s > 0.05).

### Results Based on the Cut-off Scores

At post-intervention, 37.3% of participants’ PTSD scores remained above the cutoff (from 100% at pre-intervention), and only 8.8% of participants’ scores were above the cutoff at the one-month follow-up assessment.

### Qualitative Results from Participant Feedback and Facilitator Interviews

Participants described the group intervention as a pleasant and helpful experience. For example: “Here I have learned how to remove the disturbing memories of the attack from my mind- a 12 years old participant”, in another participant’s words *“*I am very satisfied with the sessions, they helped us overcome what have been bothering us; I thank all the psychologists- a 13 years old girl”. Participants also noted that the group intervention provided them with the opportunity to meet other individuals who shared their feelings “I was scared of talking about the attack, here I could talk about it with my groupmates, I learned to listen to others and I felt good when I spoke out my mind- a 12 years old participant”, talk about the tragic attack and its effects on them “I was terrified that the attack would happen again, I was afraid going back to school, I was always deeply sad [Jigarkhon/Extremely sad]- a 10 years old participant”, and receive help from the group facilitators “Our teachers [group facilitators] have been very kind, they helped me learn how to calm my body when I am scared and what to tell myself when bad things come to my mind- a 13 years old participant”.

Some of the participants expressed a desire for more sessions, and all of them appreciated the engagement, patience and the techniques taught by the group facilitators “I have learned how to overcome my fears, regain my self-confidence and hope for the future, thanks to our teachers [group facilitators], they have been kind and diligent, I wish we had more sessions- a 12 years old participant”. 

Participants' written feedback also indicated improvements in specific areas of their lives “I can better concentrate on my lessons, I want to get the highest grade in my class- a 12 years old participant”, “I am more kind to my classmates- a 10 years old participant”, “I learned to talk with my mom when I am worried, I learned to cry when I want to, it is not bad to cry- a 10 years old participant”, their psychological wellbeing “Now I can recognize when I am scared, angry or sad, and I know what to do about them- a 15 years old participant” or as another 13 years old stated “My worries, breath shortness and distraction have been cured”, and particularly the problems stemming from exposure to the terrorist attack “I realized that after attending the sessions, I am not fighting and arguing with my parents and siblings, what they say does not irritate me now- an 11 years old participant” or “I am not scared of looking at the entrance of our school – a 16 years old participant” or “I take a deep breath when I am angry – a 13 years old participant”.

The vast majority of children and adolescents indicated that the core benefit of the intervention was the normalization of the harsh experience of the terrorist attack, for example: “I noticed that what happened was terrifying to everyone- a 15 years old participant” or “I learned that my headache, forgetfulness, sadness, and irritability were because of the attack, and most of my groupmates have the same symptoms- a 16 years old participant”. They also noted that the group intervention taught them techniques to cope with the disturbing consequences of the terrorist attack “In these classes, I learned how to manage my fear- a 13 years old participant” or “I learned the techniques to work on my fear- a 14 years old participant”, “I learned to work on my nightmares, now I am writing them like a story with a positive ending- a 16 years old participant”. Participants reported that the intervention helped them overcome “*Tars”*- fear of and unwillingness attending school and “*Negrani”*- which refers to anxiety and worry. Problems related to the aftermath of the terrorist attack were also reduced. Participants mentioned that their nightmares with traumatic content, reminders of the attack, and intrusive thoughts and images pertaining to the incident had disappeared by the end of the sessions. For example, “Before these sessions, I was an [Jigarkhon]-extremely sad person, but now I am happy and when something related to the attack comes to my mind, I use the dual attention techniques and think of good memories”.

Participants also reported improvements in their physical health conditions and bodily symptoms. Suffering from headaches and loss of appetite were prevalent complaints among the participants at the beginning of the program. By the end of the intervention, they reported improvements in these areas as one of them noted “After the attack, I had no appetite, I had constant headaches and sleep problems, but now they are resolved”.

The intervention improved the participants’ relationship with their friends and family members, helping them feel closer and more appreciative of their presence. Some of the participants attributed this change to their learning about types of emotions, how they manifest and how to regulate them, as one of the participants expressed “Before attending the sessions, I was always angry at everything, but now I know how to recognize my emotions and how to deal with them”.

Participants reported a positive effect of the intervention on their hopefulness, resilience, and optimism toward the future. They expressed their appreciation for life, their friendships, the support they received from their families and helpers. One of the participants stated “I want to continue my education and enjoy my life. I am sad about my friends who died in the attack, but I have learned to take care of myself and pray for them. I want to become a doctor”.

The experience of delivering the program was perceived as empowering and educative for the facilitators. The quality of the program, timely supervision, and on time logistical support appear to have been tangible for the group facilitators. The facilitators learned about assessment in emergency settings, trauma recovery techniques, and how to pay attention to the diverse individual experiences in the groups.

## Discussions

The aim of this study was to investigate the potential of the Children and Grief (C&G) program in alleviating PTSD symptoms and enhancing psychological wellbeing in Afghan (Hazara) children and adolescents affected by a terrorist attack on a school in Afghanistan. Overall, PTSD symptoms decreased and the findings are in line with previous research indicating the effectiveness of the C&G in reducing Post-Traumatic Stress and Traumatic Grief symptoms (Barron & Abdallah, 2017), as well of the studies that evaluated the efficacy of the TRT program - that is the first 5 sessions of the C&G program, in occupied Palestine and Brazil for violent military occupation and drug violence respectively (Barron et al., 2013; Barron et al., 2021).

Both post-intervention and one-month follow-up assessments indicated significant reductions in post-traumatic stress symptoms and psychological distress. Similarly, for intrusion and avoidance symptoms, the reductions remained statistically significant at post-intervention and at one-month follow-up assessments. This is a noteworthy finding, especially considering the cumulative stress and potential intergenerational trauma among the study’s population and the ongoing uncertainty in the lives of these children and adolescents.

The group-based C&G program delivered as a low intensity intervention provided access to psychological support for a substantial number of children and adolescents. This is particularly important in contexts with high demands and limited resources to access mental health services (Mohd Saleem et al., 2021; Patel et al., 2008). This finding is in line with Dyregrov et al. (2011) and suggests that the C&G program has the potential to reduce the need for individualized treatments.

The program was delivered by trained local resources (psychology graduates and final-year psychology students) with no prior experience of working with large groups of highly affected individuals. Nevertheless, facilitators reported that the program was convenient to implement and associated with reduced stress, citing the clear structure of the protocol and the comprehensive explanations provided in the manual. Participants, on the other hand, found it engaging to begin with practical techniques such as *“Creating a Safe Space”* and the use of *“The Feeling Thermometer”.* The use of familiar terminology and child-friendly names for the techniques, together with step-by-step instructions that did not require advanced reading or writing skills, greatly supported students’ understanding of the techniques and their ability to follow the instructions. This, in turn, led to increased treatment adherence with improved engagement, willingness to disclose, and decreased symptoms. The level of education that an intervention requires, and the chosen techniques are important when an intervention is implemented in certain contexts. For example, in a study aimed at evaluating “modified-written exposure therapy” with adolescents who shared language and traumatic experiences similar to those of the current study’s target group, Ahmadi et al. (2022b) found that some participants wrote less due to a lower level of education.

Participants also emphasized the benefits of psychoeducation and the normalization of their experiences, as well as learning techniques for managing intrusive thoughts and memories, practicing body relaxation, using powerful self-talks, and setting goals for the future. Similarly, the program was reported as highly relevant and effective by the group facilitators.

The first adaptation in this study involved delivering the instructions in the community’s dialect (Hazaragi) when needed. Hazaragi is a derivative of the Persian Dari language, with slight differences in accent and some unique expressions. Participants reported that it was helpful that the instructions were tailored to their level of knowledge and to the specific subcultural context to which they belong. This seems particularly crucial when working with a target group that has a history of suppression based on factors such as ethnicity, religious beliefs, or physical appearance. In this regard, Skrodzka et al. (2020) in a study on the Lemko ethnic minority in Poland found that minority language use acts as a buffer against behavioral and emotional symptoms of historical trauma, presumably by providing an opportunity for expressing traumatic experiences, acquiring social supports, connecting with other community members, and maintaining a sense of cultural continuity and emotional attachment to ancestral territory. Moreover, a meta-analysis indicated that interventions tailored to accommodate individuals’ cultural perspectives on their illness yield the most positive outcomes (Benish et al., 2011). Participants exhibited greater engagement with the intervention when they felt their dialect was acknowledged and valued. This recognition was found to relieve them from the struggle of expressing their feelings due to linguistic challenges. Participants reported a sense of ease and freedom when discussing their thoughts in their native dialects, without worrying about articulating their feelings.

The second adaptation to program delivery involved providing extra leisure activities to children and adolescents during the sessions’ breaks, for example, allowing them to read storybooks or engage in drawing. Future studies can compare the efficacy of delivering the C&G program with a break within sessions and without a break to assess if this socialization has an effect on the treatment outcomes. Thirdly, since the terminology for naming and defining emotions was found to be unclear for participants, special attention was given to teaching about feelings and emotional expressions. This training, as outlined in the intervention manual, assisted participants in identifying various emotions triggered by reminders of the event, labeling these emotions, and differentiating between typical and distressing levels of emotional responses linked to the terrorist attack. Local expressions were used to synonymize the names of emotion, enhancing clarity, while maintaining the integrity and categorization of the manual’s content. Fourthly, given that the main complaints among participants were primarily related to the impact of the terrorist attack, the main focus of the intervention was centered on alleviating PTSD symptoms rather than grief. Consequently, certain practices such as “Symbolic Burial ceremony”, “Creation of memory box for collecting reminders of deceased”, and “Addressing long term grief” were not included in the intervention. The intervention did, however, incorporate elements aimed at overcoming feeling of guilt, remembering those who had passed away, and praying for them in line with culturally relevant practices.

Although a family orientation session was offered for parents of the participants prior to the intervention, due to imposed restrictions on gatherings by the de facto authorities, the planned sessions for the parents could not take place. Later when participants’ feedback were collected, it seemed that additional family training was required to enhance the effect of the intervention and educate the parents about their reaction and those of their children and adolescents following the terrorist attack. Studies also suggest the need to raise awareness about mental health issues in Afghanistan and recommend active engagement of teachers and family elders (Oriya & Alekozai, 2022).

In contrast to a school-based intervention in Afghanistan, where limited teacher supports for releasing students from classes led to higher dropout rates (Ahmadi et al., 2023), the present study benefited from a high level of cooperation from both school authorities and teachers. Several factors may have contributed to this positive attitude. First, as the intervention was part of a bigger project, multilateral engagement was secured prior to implementation and included school authorities and community leaders. Engagement of the public and stakeholders is essential throughout the process from development to implementation, ensuring a focus on addressing local needs and maintaining participant motivation over time (Giebel et al., 2024). Second, the project provided the school with additional support, such as sport equipment, stationery and medication which may have further encouraged cooperation. This finding underscores the significance of effective coordination with local communities to ensure that all involved bodies receive comprehensive information about the intervention (IASC, 2021). Both seamless coordination and integrated interventions as suggested good practices in interventions and support in major emergencies (Williams et al., 2007), appeared to play a pivotal role in promoting treatment adherence and fostering institutional cooperation.

Given the cultural sensitivity and the restrictions imposed after the government’s collapse, groups were separately formed for girls and boys. With the robust support of community leaders (*Shura*), girls who had been affected by the terrorist attack and enrolled beyond the sixth grade, but unable to attend school due to the ruling regimes’ restrictions, were included in the intervention. This further highlights the essential role of coordination and community engagement in research and service provision.

The C&G program was implemented in an exceptionally challenging emergency. The intervention coincided with the collapse of the former Islamic Republic of Afghanistan government and the Taliban’s takeover. While the assessment, allocation to the groups and introductory sessions with the caregivers had been completed before the change in political power, the presentation of the program had not yet started. The intervention was suspended for approximately two months due to the suspension of all public and private institutions. Massive restrictions had been imposed that directly affected the ongoing intervention. The previously contracted safe places were closed, and some of the beneficiaries left the country. Adolescents and children affected by the terrorist attack found themselves in yet another stressful circumstance as they were banned from attending school and faced potential threats. The current study suggests that the challenge of a situation should not serve as a hindrance to researching and developing feasible interventions for adolescent in war-torn, fragile, and humanitarian settings. It is essential that psychological research is conducted in humanitarian contexts in low-income countries, even if there are challenges inherent in these contexts (Ahmadi et al., 2023).

## Limitations

The study was a pre-post design without randomization or a control group, mainly because assigning highly affected individuals to a control group raised ethical concerns and was not feasible given the contextual requirements. Therefore, the results are promising yet preliminary. Participants were purposively selected to include children and adolescents with significant levels of post-traumatic stress. This selection was unavoidable given the context in which the program was implemented. In effect, large F-ratios were obtained, this may partly reflect regression towards the mean, whereby extreme values in the initial measurement tend to move closer to the center of distribution for a later measurement (Davis, 1976; Lin & Hughes, 1997). Response style or extreme responding to measurements (Liu et al., 2017) which was not handled in this study could also partly explain the effects observed. However, even after considering factors such as regression to the mean and extreme responding, the written feedback consistently highlighted improvements, suggesting the presence of meaningful change.

This study did not address the role of grieving for classmates or schoolmates, which could potentially influence how children and adolescents cope with aftermaths of trauma combined with grief symptoms. Validity and reliability of the Persian Dari version of the measures included in this study were unknown. The study did not assess additional comorbid psychological problems, adverse childhood experiences (ACEs), and other trauma types beyond PTSD and general psychological distress, primarily due to the emergency condition, the need to prioritize brief assessments, and the urgency of intervention delivery. We suggest that future research in similar settings account for these variables and investigate their associations with the efficacy of interventions for individuals exposed to prolonged conflict and violence. Moreover, the data for PTSD subscales from 30 individuals were inaccessible due to inadequate documentation, the overall results regarding intrusion and avoidance symptoms should be interpreted noting this limitation.

Lastly, the study’s target group were the 10–17-year-old Hazara children and adolescents living in Kabul. Most were female. It is important, therefore, to be cautious when generalizing the results. There may be significant differences in terms of culture and living conditions as well as gender and age among children and adolescents.

## Conclusions and Implications

The findings suggest that the C&G program, when delivered in a group format, is a promising intervention for reducing psychological distress among children and adolescents affected by a terrorist attack in a school setting. The results also indicate that the program can be delivered by trained non-mental health professionals and it is effective in an active conflict context such as Afghanistan.

As the results of the study are promising, it is recommended that the C&G program continue to be used for children and adolescent population in Afghanistan in response to terrorist attacks. It is premature, however, to recommend that the C&G program is efficacious for other emergency and war-related events and for children under 10 years of age.

Given the promising results of an uncontrolled study, it is recommended that a randomized controlled trial be conducted for children in Afghanistan who have received the C&G program following a terrorist attack. The sample size should be calculated to ensure sufficient power for the study, and the validity and reliability of the Persian Dari measures should be evaluated. Similar numbers of males and females should make up the sample population, and the program should be tested with children younger than 10 years as well.

## Data Availability

The de-identified, anonymized data supporting the findings of this study are available from the corresponding author upon reasonable request.
